# Analysis of the Proteolytic Processing of ABCA3: Identification of Cleavage Site and Involved Proteases

**DOI:** 10.1371/journal.pone.0152594

**Published:** 2016-03-31

**Authors:** Nicole Hofmann, Dmitry Galetskiy, Daniela Rauch, Thomas Wittmann, Andreas Marquardt, Matthias Griese, Ralf Zarbock

**Affiliations:** 1 German Centre for Lung Research, Dr. von Hauner Children’s Hospital, Ludwig-Maximilians University, 80337, Munich, Germany; 2 Proteomics facility, University of Konstanz, 78547, Konstanz, Germany; University of S. Florida College of Medicine, UNITED STATES

## Abstract

**Rationale:**

ABCA3 is a lipid transporter in the limiting membrane of lamellar bodies in alveolar type II cells. Mutations in the *ABCA3* gene cause respiratory distress syndrome in new-borns and childhood interstitial lung disease. ABCA3 is N-terminally cleaved by an as yet unknown protease, a process believed to regulate ABCA3 activity.

**Methods:**

The exact site where ABCA3 is cleaved was localized using mass spectrometry (MS). Proteases involved in ABCA3 processing were identified using small molecule inhibitors and siRNA mediated gene knockdown. Results were verified by *in vitro* digestion of a synthetic peptide substrate mimicking ABCA3’s cleavage region, followed by MS analysis.

**Results:**

We found that cleavage of ABCA3 occurs after Lys^174^ which is located in the proteins’ first luminal loop. Inhibition of cathepsin L and, to a lesser extent, cathepsin B resulted in attenuation of ABCA3 cleavage. Both enzymes showed activity against the ABCA3 peptide *in vitro* with cathepsin L being more active.

**Conclusion:**

We show here that, like some other proteins of the lysosomal membrane, ABCA3 is a substrate of cathepsin L. Therefore, cathepsin L may represent a potential target to therapeutically influence ABCA3 activity in ABCA3-associated lung disease.

## Introduction

ABCA3 is a member of the subclass A of the large ABC transporter family which comprises transporters involved in cellular lipid transport [1]. ABCA3 is strongly expressed in the lungs where it localizes to the outer membrane of lamellar bodies (LBs) in alveolar epithelial type II cells [2,3]. It transports phospholipids and cholesterol into the LB lumen and is essential for the biogenesis of LBs [4,5]. Mutations in ABCA3 cause an often fatal severe respiratory distress syndrome in new-borns and diffuse parenchymal lung disease in children (chILD) [6,7].

To date, little is known about the cell biology of ABCA3. After folding in the ER and glycosylation in the Golgi apparatus, ABCA3 is trafficked to the endosomal compartment and finally reaches acidic, lysosome-derived multivesicular bodies, precursors of LBs [8]. The exact route ABCA3 takes remains elusive; for example, it is currently unknown whether it passes the plasma membrane. Interestingly, in immunoblots ABCA3 gives two protein bands with an apparent molecular mass of approximately 190 and 170 kDa, respectively [4,9]. We showed previously that the lower band arises by proteolytic cleavage at the N-terminus of ABCA3 [10]. In the same study, we also identified post-trans-Golgi acidic vesicles as the intracellular compartment of ABCA3 processing and provided evidence for the involvement of a cysteine protease.

Since it can be expected that cleavage of ABCA3 has an effect on the protein’s function, the protease(s) involved the processing of ABCA3 represent a potential therapeutic target. Inhibition of the enzyme(s) in order to elevate the amount of ABCA3 may counteract diminished ABCA3 activity as a result of mutations or diminished expression due to disturbed gene regulation. Therefore, the objectives of the present study were the identification of the protease(s) cleaving ABCA3 and of the precise cleavage site.

## Materials and Methods

### Cell Culture

A549 cells were obtained from DSMZ (Braunschweig, Germany). Cells were maintained in RPMI 1640 medium (Life technologies, Darmstadt, Germany) supplemented with 10% FBS at 37°C and 5% CO_2_. Stable transfection of A549 cells with *pUB6-ABCA3-WT* vector was carried out as previously described [11]. Cleavage site mutations were introduced into *pUB6-ABCA3-WT* using the Q5 Site-Directed Mutagenesis Kit (NEB, Frankfurt/Main, Germany) according to the manufacturer’s instructions. For inhibitor experiments, cells were grown to confluence, trypsinized and seeded at 200.000 cells per 6-well and grown for 48 h prior to treatment. For siRNA mediated knockdown, cells were trypsinized and cell suspension was adjusted to 200,000 cells / ml in RPMI medium with 10% FBS. 2 ml of cell suspension was then added to a mixture of siRNA (125 pmol / well; Life technologies) and Lipofectamine 2000 (8 μl / well; Life technologies) in OptiMEM (Life technologies) dispensed in 6-well plates. Cells were harvested after incubation with siRNA for 48 h. Scrambled siRNA (Life technologies) was used as control.

### Gel Electrophoresis and Immunoblot

After harvesting by trypsination, cells were rinsed with PBS once and subsequently lysed with radioimmunoprecipitation (RIPA) buffer (0.15 M sodium chloride, 1% Triton-X 100, 0.5% sodium deoxycholate, 0.1% SDS, 5 mM EDTA and 50 mM Tris pH 8) containing complete protease inhibitor (Roche, Mannheim, Germany). The lysate was centrifuged for 30 min at 1000 x g and 4°C. The protein concentration of the post-nuclear supernatant (= whole cell lysate) was determined with Bradford assay using BSA as protein standard. 15–30 μg of cell lysates in 4x LDS buffer (Life technologies) were loaded onto NuPage Mini Bis-Tris or Tris-Acetate gels (Life technologies). Following gel electrophoresis, proteins were visualized using Coomassie Brilliant Blue (Sigma-Aldrich, Steinheim, Germany) or transferred to PVDF-membranes (Millipore, Billerica, USA) and immunoblotted using anti-HA-tag (Roche) and anti-β-actin HRP conjugate (Santa Cruz, Heidelberg, Germany). Chemiluminiscent signal was detected by ECL Detection Reagent (GE Healthcare, Freiburg, Germany) and analyzed by densitometry.

### RNA Isolation/cDNA Synthesis/Quantitative Real Time PCR

Cells grown to confluence in 6-well plates were washed once with PBS. Cells were harvested and total RNA was isolated with the High Pure RNA Isolation Kit (Roche, Mannheim, Germany) according to the manufacturer’s instructions. RNA concentrations were measured with a NanoDrop spectrophotometer (Thermo Scientific, Waltham, MA, USA). 1 μg of total RNA was reversely transcribed into cDNA with the Tetro reverse transcription kit (Bioline, Luckenwalde, Germany). Quantitative real-time PCR was carried out using SensiFAST SYBR Hi-ROX Mix (Bioline) on an ABI 7900HT cycler (Applied Biosystems, Darmstadt, Germany). HPRT1 was used as housekeeper gene. For analysis of relative changes, data was analyzed according to the ΔΔC_T_ method.

### In-gel Digestion and Peptide Extraction

Gel bands visualized with Coomassie Blue were excised, destained and digested with trypsin gold mass spectrometry grade (Promega, Madisson, USA) according to the manufactures’ protocol. Extracted peptides were subjected to LC-MS/MS analysis.

### Liquid Chromatography-Tandem Mass Spectrometry (LC-MS/MS) and Data Analysis

Peptide mixtures were separated using an Eksigent nano-HPLC (Eksigent Technologies, Dublin, USA) with a reversed-phase LC column (5 μm, 100 Å pore size C18 resin in a 75 μm i.d. × 10 cm fused silica capillary; Acclaim PepMap100; Thermo Scientific, Waltham, USA). After sample injection, the column was washed for 5 min with 95% mobile phase A (0.1% formic acid) and 5% mobile phase B (0.1% formic acid in acetonitrile), and peptides were eluted using a linear gradient of 5% mobile phase B to 40% mobile phase B in 65 min, then to 80% B in an additional 5 min, at 300 nL/min. Mass spectrometric analysis was performed on an LTQ-Orbitrap Discovery mass spectrometer (Thermo Fisher Scientific, Bremen, Germany) operated in a data dependent mode in which each full MS scan (*m/z* 200–1,450) acquired in Orbitrap with resolution of R = 30,000 (*m/z* 400) was followed by five MS/MS scans where the five most abundant molecular ions were dynamically selected and fragmented by collision-induced dissociation (CID) using a normalized collision energy of 35% in the LTQ ion trap. Dynamic exclusion was allowed.

Mascot database searching was performed using the Mascot Server 2.3 software (Matrix Science, London, UK); all tandem mass spectra were searched against the SwissProt and NCBInr human protein databases. Search criteria for tryptic digest mixtures included trypsin as an enzyme, one missed cleavage, methionine oxidation as variable modification and mass tolerances of 5 ppm for MS and 0.5 kDa for MS/MS. The results were filtered to a 5% maximal false discovery rate; peptide score cut-off of 15 was used. Identification of modified and non-specifically cleaved peptides was additionally performed by the Mascot error tolerant search. Presence/absence of ABCA3 sequence parts ware validated with Xcalibur software (Thermo Finnigan, San Jose, CA, USA) using extracted MS ion chromatograms of the accurate peptide *m/z* values ±5 ppm related to corresponding peptide ions.

### *In Vitro* Analysis of Products from Cleavage of ABCA3 with Cathepsins B and L

Synthetic KYHLRFSYTRRNYMWTQTGSFFLKETEGWHTTSLFPLFPNPGPR peptide (HPLC purity 98%), comprising residues 151–194 of human ABCA3, was purchased from GenScript (Piscataway, USA). Peptide cleavages were performed with cathepsin B purified from human placenta (Sigma-Aldrich) and human recombinant cathepsin L (GeneTex, Irvine, USA) using single cathepsins or their combination. Peptide and cathepsins were mixed in 100 μL 10 mM ammonium acetate pH 4.5 to a final concentration of 20 μM for the peptide and 2 μM for either of the cathepsins. After 20 min incubation at 37°C samples were immediately analyzed by LC-MS/MS.

### Statistics

Comparisons of multiple groups were done using one-way repeated measure ANOVAs with Tukey’s post hoc test. Results were presented as mean + S.E.M. of a minimum of three different experiments. P-values of less than 0.05 were considered statistically significant. All tests were performed using GraphPad Prism 5.0 (GraphPad Software, La Jolla, USA).

## Results

### Mass Spectrometric Analysis Reveals N-Terminal Cleavage Region of ABCA3

**Fig 1:**
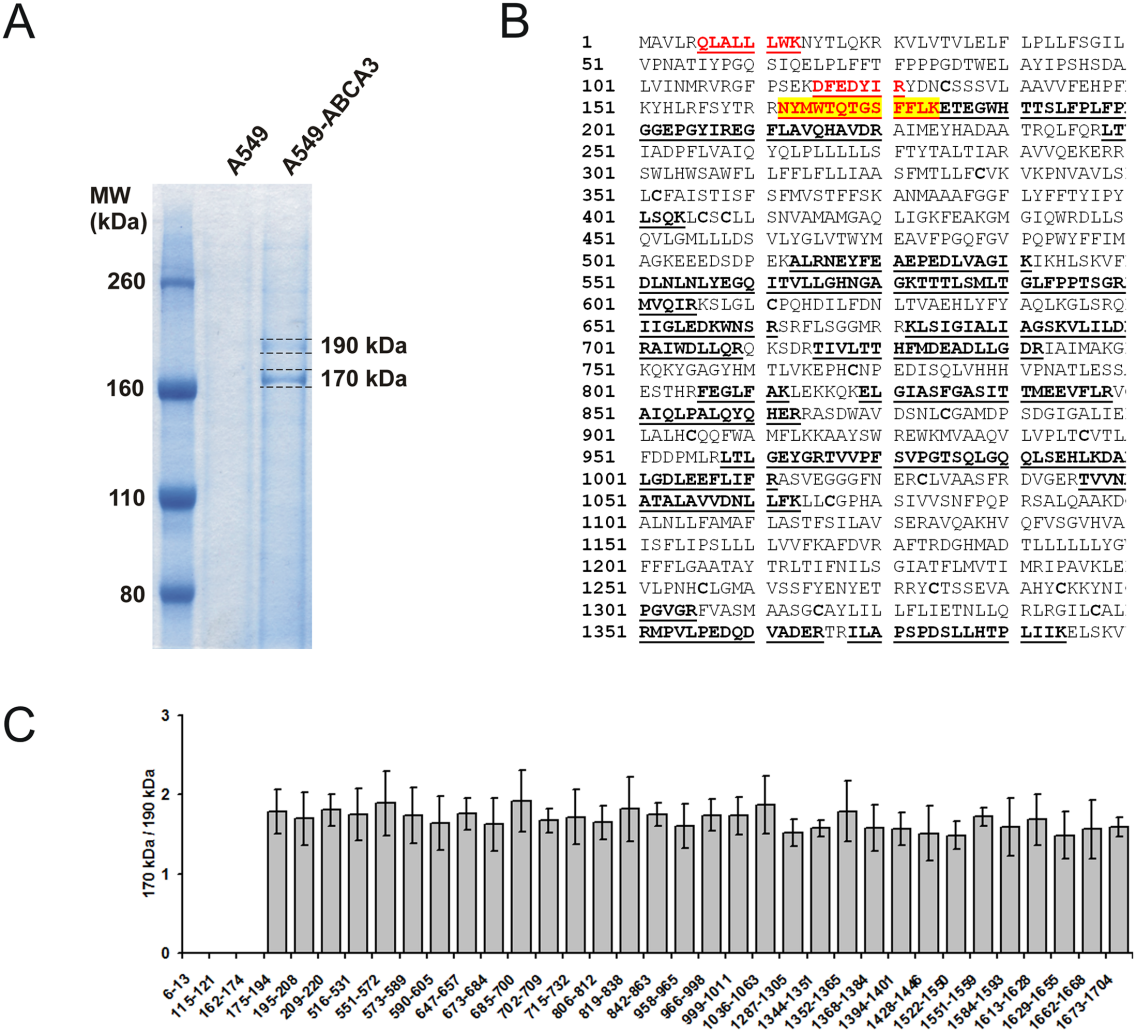
Analysis of ABCA3 cleavage region. **A**: ABCA3 containing gel bands were excised, digested with trypsin and analysed. **B**: Partial sequences of unprocessed (190 kDa) and processed (170 kDa) ABCA3 forms identified by LC-MS/MS. Identified sequence parts of ABCA3 precursor protein including HA-TAG with spacer (shown in blue) are underlined. Sequences identified only in unprocessed form and absent in processed form are shown in red. Cleavage region (162–174) is highlighted in yellow. **C**: Peptide intensity ratios from MS ion chromatograms (three measurements), leading to the predicted cleavage region.

**Fig 2:**
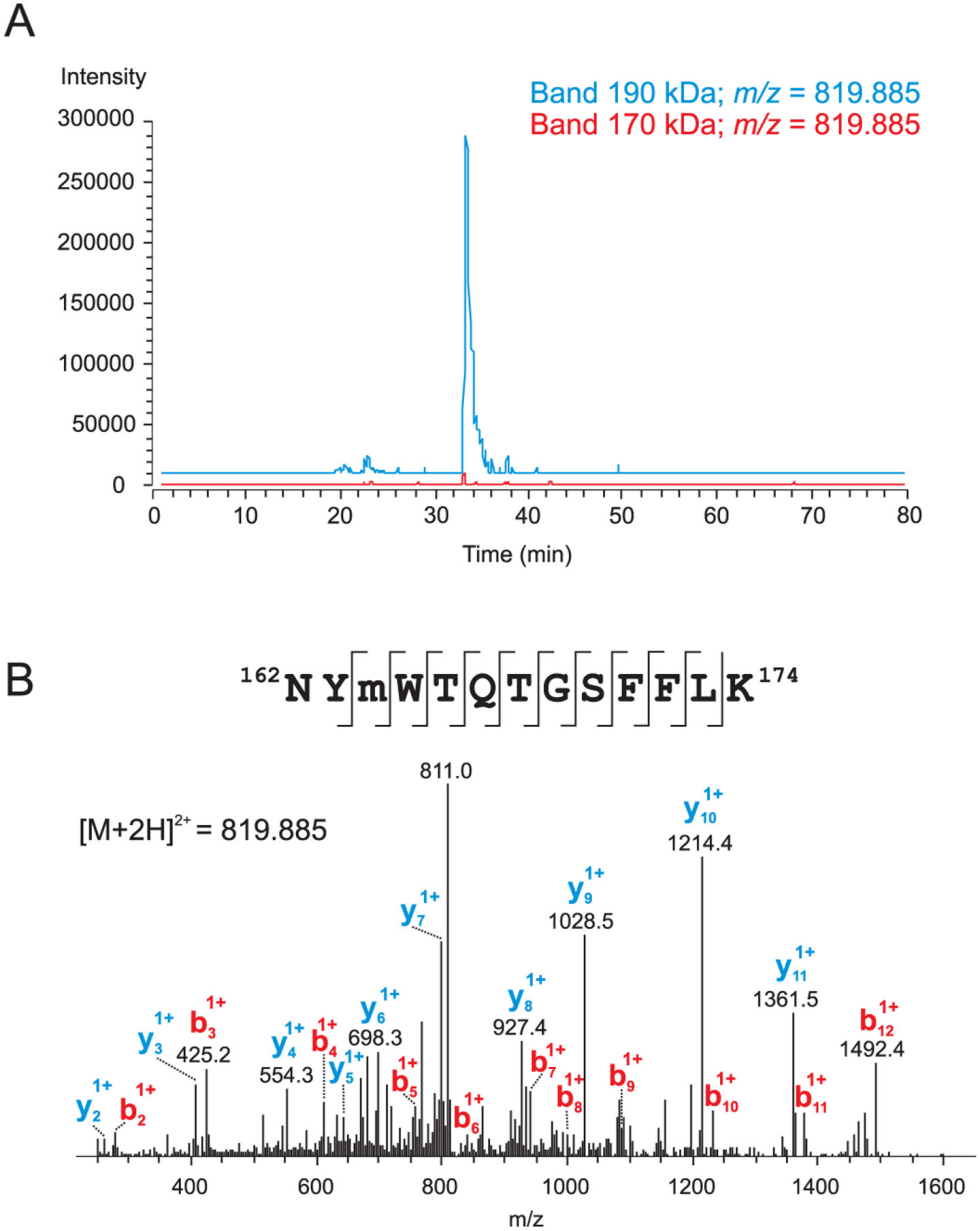
Partial sequence (162–174) is present in unprocessed ABCA3 and missing in processed protein form. **A**: Intensity profiles of an *m/z* range corresponding to the monoisotopic peak of the doubly-charged ion at *m/z* 819.885 (*m/z* window ±5 ppm) for 190 kDa and 170 kDa gel bands acquired in Orbitrap. **B**: Identification of the ABCA3 tryptic peptide comprising amino acid residues 162–174 of the precursor protein and containing methionine sulfoxide at position 164. MS/MS spectra after CID fragmentation measured in the LTQ ion trap are shown. Major *b* and *y* ions are labelled on the spectra and all fragment ions obtained are marked on the identified sequence. The ion at *m/z* 811 corresponds to the loss of water from the parent ion.

Protein cleavage site or region can be quantitatively determined using intensity ratios of tryptic peptides or their fragments in cleaved and non-cleaved protein forms [12]. We separate proteins of A549 cells transfected with *pUB6-ABCA3-WT* vector by gel electrophoresis, visualized them with Coomassie Blue, and analyzed by LC-MS/MS. ABCA3 protein was identified in gel bands at 190 kDa and 170 kDa (Fig 1A). Profile analysis using highly accurate (±5 ppm) MS chromatograms for corresponding peptide ions revealed the presence of the C-terminal protein part comprising residues (175–1704) in both bands. Tryptic peptides from the N-terminal protein region (6–174) were detected only in the upper band. Parts of the ABCA3 protein sequence identified in 190 kDa and 170 kDa gel bands are presented in Fig 1B. Fig 1C illustrates the quantitative MS approach used for the determination of the cleavage region. The peptide intensity ratios (170 kDa / 190 kDa) was 1.7±0.4 within the sequence (175–1704) and ~0 for the peptides (6–13), (115–121) and (162–174) from the N-terminal region (Fig 1C). Remarkably, highly intense signals of doubly-charged ions at *m/z* 819.885 and at *m/z* 811.887 identified as peptide ^162^NYMWTQTGSFFLK^174^ with and without oxidation at Met^164^ were detected in 190 kDa band (Fig 2). These signals are completely missing in 170 kDa band. This observation provides evidence of N-terminal ABCA3 processing within the region between residues 162 and 174. Due to the relatively high signal intensity of the peptide (162–174) we would have expected the signal (X-174, X-amino acid residue after the cleavage site of protein processing) with significant intensity present only in N-terminally processed 170 kDa ABCA3. We have searched for signals with corresponding *m/z* values but could not find any. Therefore, from these experiments we propose ABCA cleavage after Lys^174^ or close to it. In this case, the peptide (X-174) is too small and lost during sample preparation or enrichment.

### Prediction of the Membrane Topology of ABCA3 Suggests that the Cleavage Site Is Located in the Vesicular Lumen

**Fig 3:**
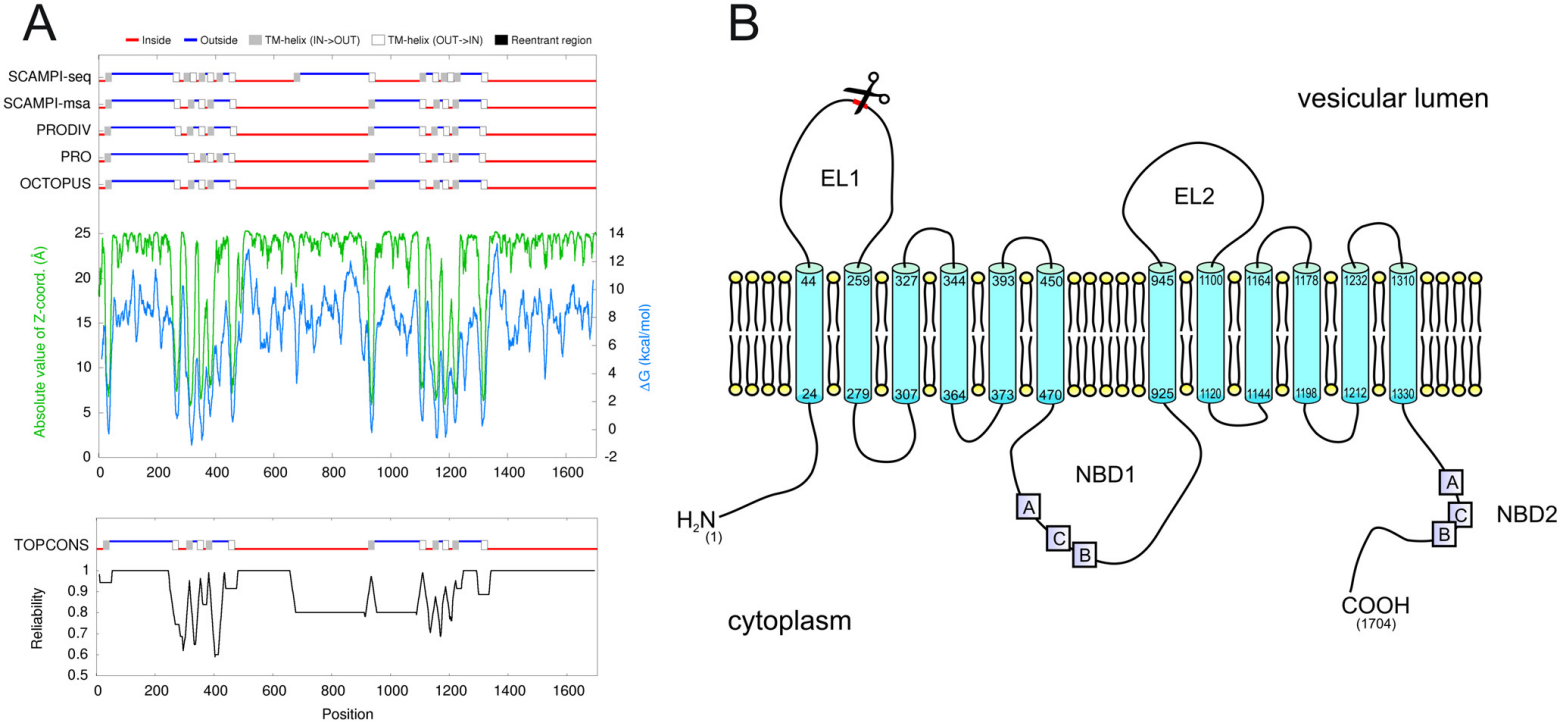
Predicted membrane topology of ABCA3 and localization of the identified cleavage site. A) Membrane topology of ABCA3 as predicted by different algorithms (top) and consensus prediction generated by TOPCONS (bottom) [13]. B) Model of ABCA3 in the vesicular membrane generated based on the TOPCONS prediction. Scissors indicate the identified cleavage site in the first extracellular loop (EL1).

We used TOPCONS (http://topcons.net/) for consensus prediction of ABCA3 membrane topology [13]. The prediction generated by TOPCONS is a consensus from five different topology prediction algorithms. The results of all five algorithms are in agreement with an outside orientation of the identified cleavage region, i.e. the region is located in the lysosomal lumen (Fig 3).

### Application of Specific Inhibitors Suggests Cathepsins L and B as ABCA3 Processing Enzymes

**Fig 4:**
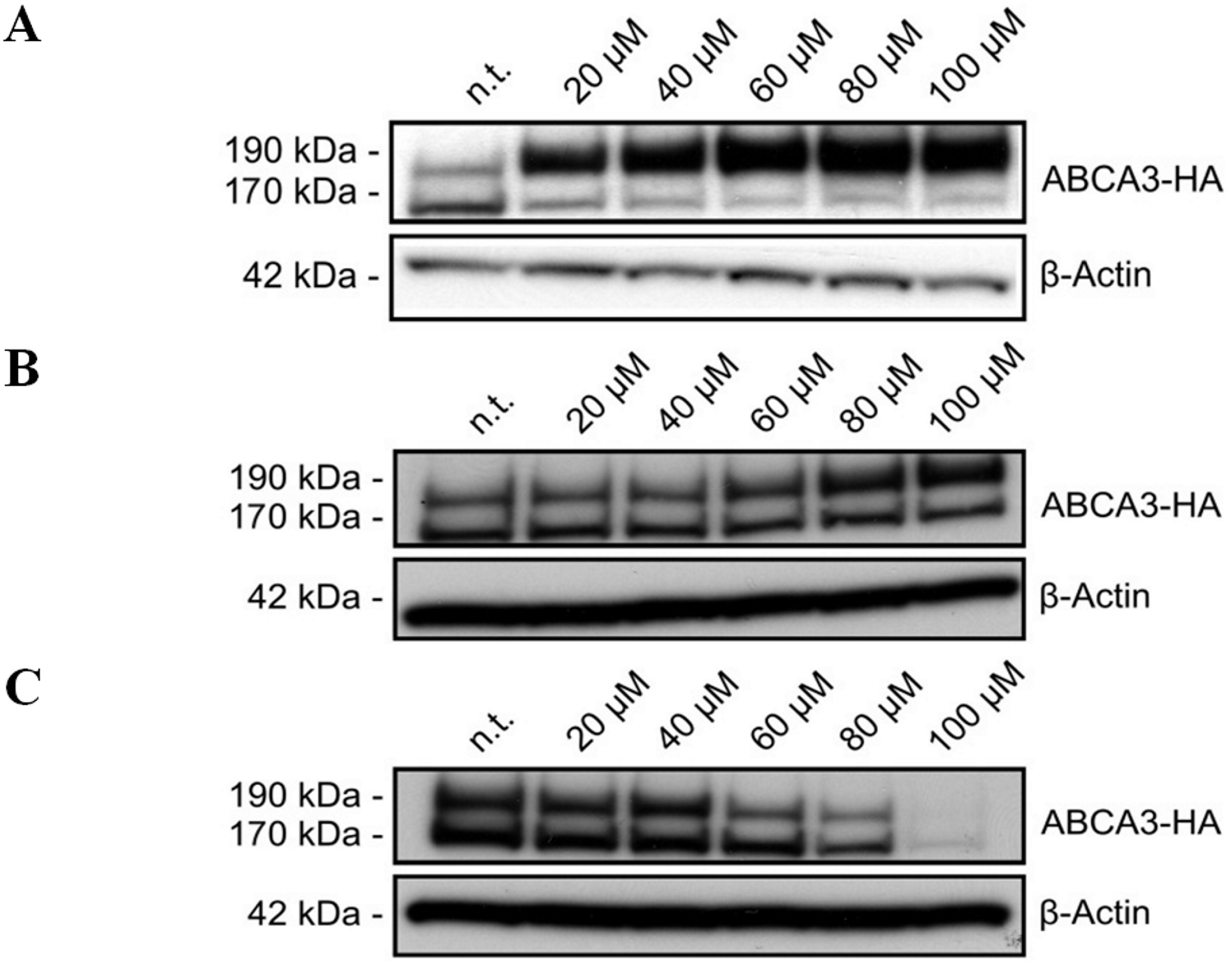
Effects of protease inhibitors on ABCA3 processing. Cells were treated with ALLM (N-Acetyl-Leu-Leu-Met-CHO) (A), CA-074 (L-trans-Expoxysuccinyl-Ile-Pro-OH propylamide) methyl ester (B), and N-(1-naphthalenylsulfonyl-L-isoleucyl-L-tryptophanal (C), and cleavage of ABCA3 was assessed by Western blotting.

From earlier studies, we knew that ABCA3 is N-terminally cleaved by a cysteine protease and that acidification of the vesicles ABCA3 resides in is necessary for cleavage to occur [10]. Based on this knowledge, we aimed to narrow down the list of proteases possibly involved by application of highly specific inhibitors. ALLM (N-Acetyl-Leu-Leu-Met-CHO) is an inhibitor of calpains I and II and also very potently inhibits cathepsins B and L [14]. ALLM almost completely abolished proteolytic processing of ABCA3 and lead to a prominent accumulation of the 190 kDa band, while the 170 kDa band became weaker (Fig 4A). Since the identified cleavage site is located in the vesicular lumen, we reasoned that the protease(s) cleaving ABCA3 would have to be lysosomally located and active at acidic pH. The latter would exclude calpains. Since results with ALLM pointed to involvement of cathepsin B and/or L, we applied inhibitors thought to be specific for cathepsin B and cathepsin L, respectively. Treatment of cells with the cathepsin B inhibitor L-trans-expoxysuccinyl-Ile-Pro-OH propylamide methyl ester (CA-074Me; [15]) resulted in accumulation of the 190 kDa ABCA3 band when concentrations of at least 60 μM were used; the 170 kDa band remained unchanged (Fig 4B). The weaker effect of CA-074Me on ABCA3 cleavage compared to ALLM suggests rather low activity of cathepsin B as an ABCA3 cleaving enzyme. For ALLM and CA-074Me, the inhibitor concentrations used did not impair the general cell growth or fitness (data not shown). Incubation of cells with the cathepsin L inhibitor N-(1-naphthalenylsulfonyl-L-isoleucyl-L-tryptophanal (NapSul-Ile-Trp-CHO; [16]) did not result in accumulation of the 190 kDa band (Fig 4C). Inhibitor concentrations exceeding 40 μM lead to a disappearing of both bands, indicating that cathepsin L inhibition with NapSul-Ile-Trp-CHO has a stronger effect on cell viability than on ABCA3 production and processing. Our results gathered using protease inhibitors thus suggest cathepsin L and—to lower extend—cathepsin B as ABCA3-processing enzymes, but the action of cathepsin L could not be proven clearly due to the adverse effects on cell viability by specific inhibition of cathepsin L.

### siRNA Mediated Knockdown of Cathepsins Confirms Involvement of Cathepsins L and B

**Fig 5:**
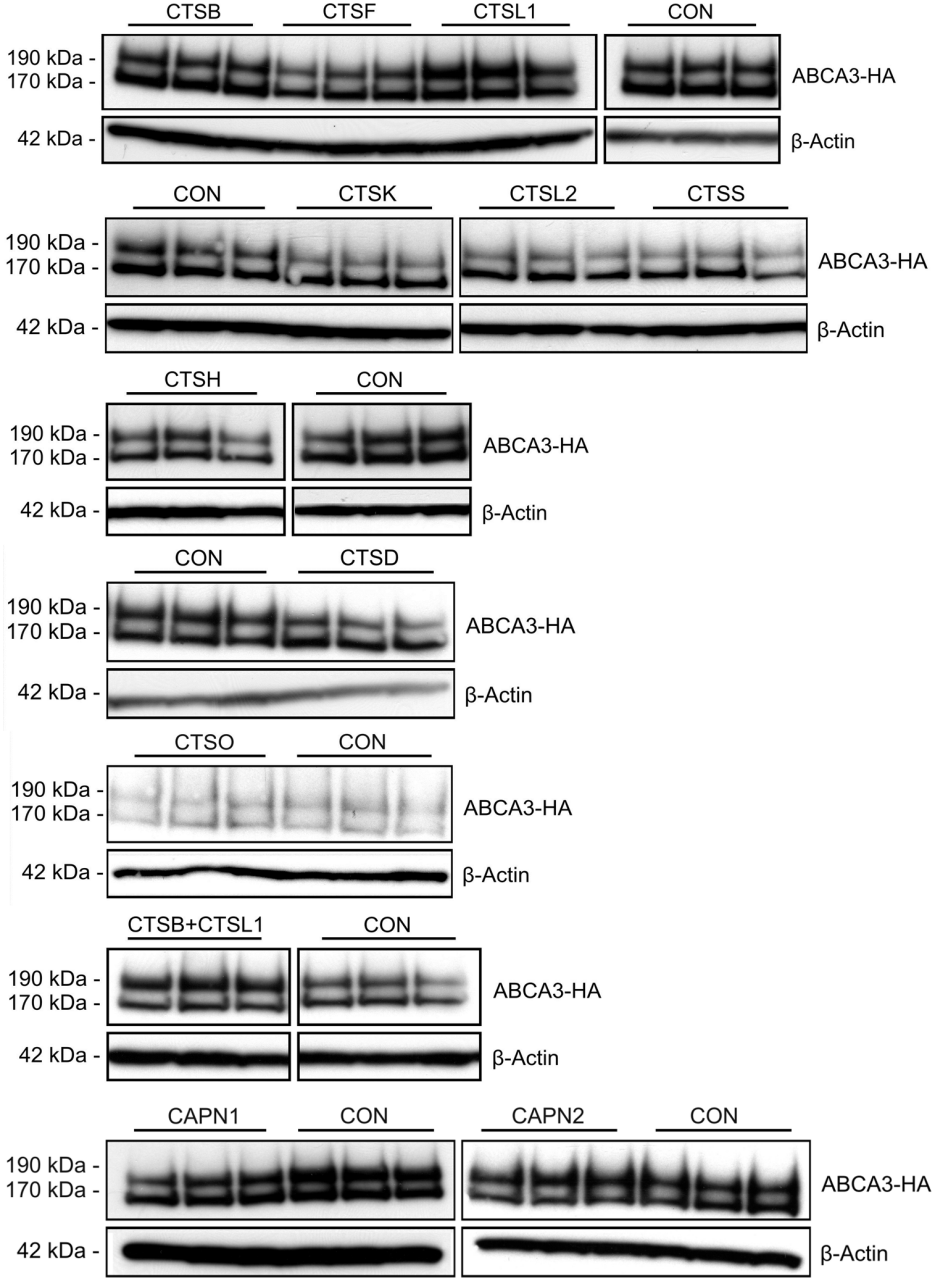
Effect of knockdown of single proteases on ABCA3 processing. Expression of lysosomal cathepsins was silenced using siRNA mediated knockdown and ABCA3 cleavage was assessed by Western blotting. Experiments were performed thrice, every time in triplicates. Representative Immunoblots are shown.

**Fig 6:**
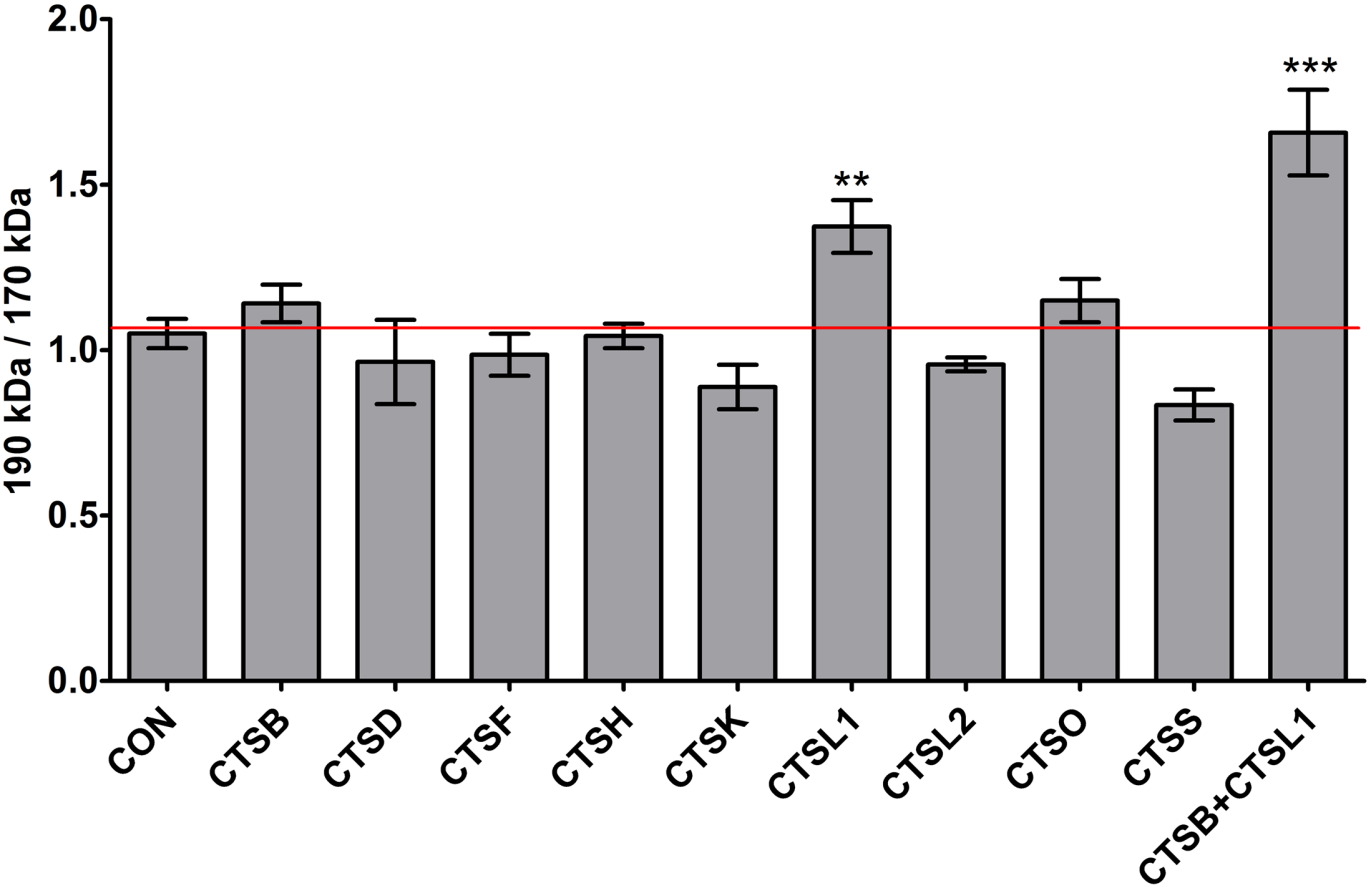
Quantitative analysis of the inhibition of ABCA3 processing. The ratio of 190 kDa to 170 kDa ABCA3 bands points to an inhibition of processing by knockdown of either CTSL1 alone or CTSL1 in combination with CTSB. Accumulation of the 190 kDa ABCA3 species is a result of inhibition of proteolytic cleavage due to cathepsin knockdown. ** p < 0.01; *** p < 0.001.

To further verify ABCA3 processing by cathepsins, we used siRNA-mediated knockdown of cathepsin genes. To do this, we applied siRNAs directed against all cysteine proteases of the cathepsin family that had been identified in lysosomes [17]. This included proteases encoded by *CTSB*, *CTSF*, *CTSH*, *CTSK*, *CTSL1*, *CTSL2*, *CTSO*, *and CTSS*. As a control, we also used siRNA knockdown of *CTSD* (Asp protease). Gene knockdown was monitored using quantitative PCR and was considered successful when expression was reduced by more than 70% (data not shown). Immunoblot performed on cell lysates collected 48 h after siRNA transfection showed accumulation of the 190 kDa band as an indicator for inhibition of cleavage only in the case of *CTSL1* (Fig 5). The effect was slightly enhanced when knockdown of cathepsin L and cathepsin B was performed simultaneously, indicating a possible additive action of both proteases. When we quantified protein bands to assess the ratio of 190 kDa to 170 kDa ABCA3 bands, we found significant increase of the band ratio in the case of *CTSL1* alone and also for the combination of *CTSL1* and *CTSB* (Fig 6). Thus, siRNA knockdown confirms the involvement of cathepsins B and L in the processing of ABCA3 and points to combined action of these proteases.

### MS Analysis of ABCA3 Peptide Cleavage Products Demonstrates Specific Cleavage after Lys^174^ by Cathepsin L

**Fig 7:**
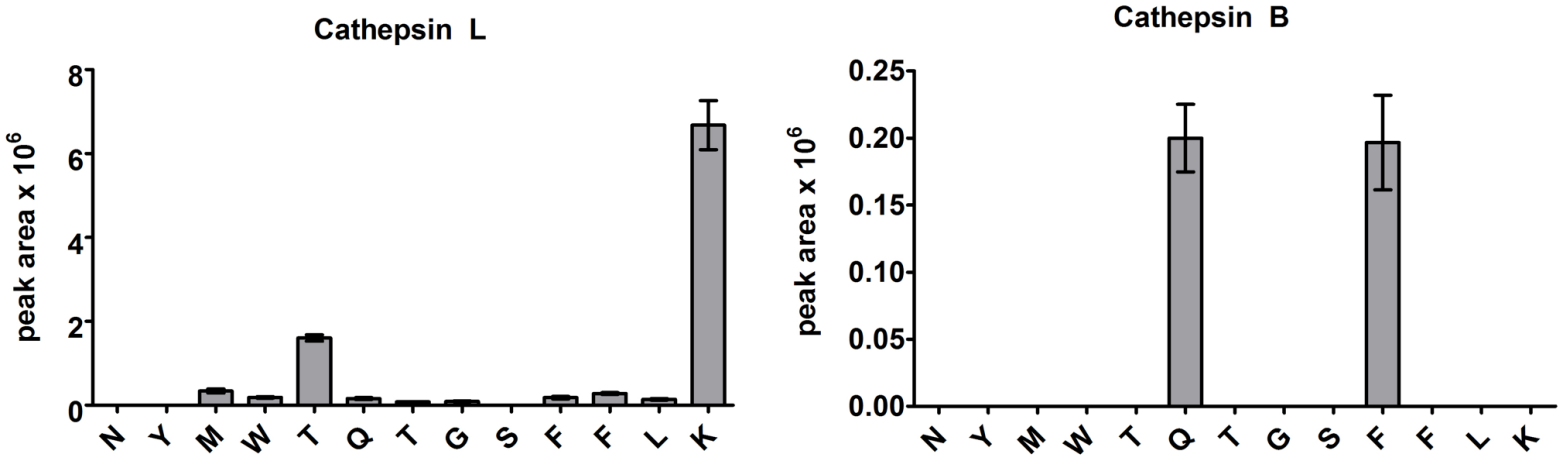
Cathepsin L cleaves ABCA3 peptide preferentially after Lys^174^. Shown are signal intensities of C-terminal cathepsins L and B cleavage products of ABCA3 peptide (151–194) within the sequence ^162^NYMWTQTGSFFLK^174^ analyzed by LC-MS/MS. Extracted MS ion chromatograms of the accurate m/z values ±5 ppm related to the corresponding peptide ions (peak areas) from three experiments were used for the diagram.

To verify the ABCA3 cleavage by cathepsins L and B, we performed proteolytic digestion of a peptide comprising residues 151–194 of ABCA3 with cathepsins L and B at acidic conditions (pH 4.5) and analyzed the cleavage products by LC-MS. Triply-charged ion at *m/z* 761.711 corresponding to the ABCA3 sequence (175–194) related to the cleavage after Lys^174^ was the most prominent signal appearing during incubation of the peptide with cathepsin L alone and in combination with cathepsin B. High cleavage specificity of cathepsin L for the peptide within the ABCA3 processing region is illustrated in Fig 7. Specificity and cleavage efficiency of cathepsin B was significantly lower in comparison to cathepsin L. Major cathepsin B cleavage products were the peptides (172–194) and (168–194) related to the cleavages after Phe^171^ and Gln^167^, respectively, in almost equivalent amounts. Cleavage patterns resulting from the combination of cathepsins L and B suggest sequential action (data not shown). Our study thus reveals highly specific ABCA3 processing by cathepsin L after Lys^174^.

### Inactivation of Putative Cathepsin L Cleavage Site Inhibits ABCA3 Processing

**Fig 8:**
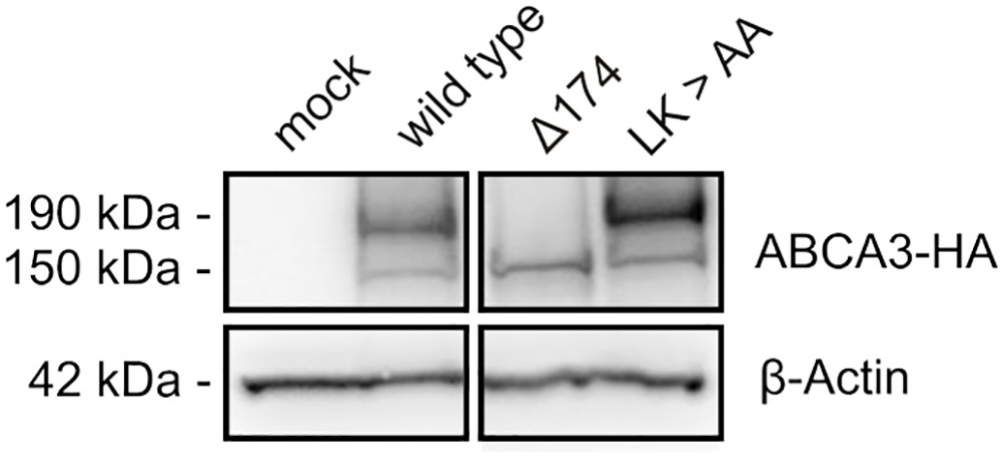
Mutation of potential Cathepsin cleavage sites. Amino acids forming potential cleavage sites were replaced by alanine residues using site directed mutagenesis. Lane 1: cells transfected with empty vector; lane 2: wild type ABCA3; lane 3: deletion of the first 174 amino acids of ABCA3; lane 4: 173LK174 > AA.

Having identified cathepsin L as the major protease responsible for cleavage of ABCA3 as well as its cleavage site, we aimed to abolish cleavage by abrogating substrate recognition. Since it is known that both amino acid residues that precede the actual cleavage site are crucial for substrate recognition, we replaced Leu^173^ and Lys^174^ of ABCA3 with alanine residues. This operation resulted in prominent accumulation of the upper ABCA3 band (Fig 8), indicating inhibition of processing and confirming correct identification of the cleavage site.

## Discussion

In the present study, we show that ABCA3 is proteolytically cleaved by cathepsin L and to a lower extent also by cathepsin B. We also identified the exact cleavage site of cathepsin L which is located after Lys^174^.

We have previously shown that ABCA3 is N-terminally cleaved in LAMP3-positive vesicles which are supposed to be lysosome-related organelles [10]. We now report identification of the precise cleavage site. According to the predicted membrane topology of ABCA3 (Fig 2), this site is located in the proteins’ first large extracellular loop. Therefore, this loop is oriented towards the acidic lumen of the vesicles and is accessible to proteases of the lysosomal matrix. This finding is in concordance with our previous report that ABCA3 is cleaved upon reaching LAMP3-positive vesicles and that cleavage is blocked by inhibition of vesicle acidification [10]. It can be speculated that the localization of the cleavage site in the largest lumenal loop of ABCA3 –which is similar to CLN7 –favours steric access for proteolytic enzymes in the lysosomal lumen [18]. The first lumenal loop also contains two N-linked glycosylation sites (Asn^124^ and Asn^140^) in vicinity of the cleavage site [19]. N-linked glycosylation of these sites in ABCA3 may limit the rate of proteolysis as it does in the cases of LAMP-1, LAMP-2, and also CLN7 [18].

We had also shown earlier that cleavage is blocked by E-64, an inhibitor of cysteine proteases. Based on these results, it was clear that the protease(s) involved in the processing of ABCA3 had to be lysosomal cysteine proteases active at acidic pH. Using a combined approach consisting of specific protease inhibitors and siRNA mediated gene knockdown, we show here that cathepsin L and B are likely involved in ABCA3 processing. However, the effect seen in the case of CA-074Me required rather high inhibitor concentrations, implicating that the specific activity of cathepsin B against ABCA3 is not very high. This assumption is corroborated by the results of *in vitro* digestion. Moreover, although CA-074 was thought to be specific for cathepsin B, it turned out that the cell permeable CA-074Me also inhibits cathepsin L to some extent [20]. Therefore, it cannot be used to reliably distinguish between cathepsin B and cathepsin L. The specific cathepsin L inhibitor NapSul-Ile-Trp-CHO prevented formation of both ABCA3 forms, a finding that is in concordance with earlier reports that NapSul-Ile-Trp-CHO causes a dose-dependent suppression of cell proliferation and cell-death in SaOS2 cells at concentrations of 40 μM [21]. It is thus possible that when treated with NapSul-Ile-Trp-CHO, A549 cells did not survive long enough to accumulate the 190 kDa ABCA3 form. Specific inhibition of cathepsin L seems to be a general problem if cancer cells are used; a strong inhibitory effect on cancer cell proliferation was reported also for other specific chemical inhibitors and neutralizing antibodies of cathepsin L [22, 23]. However, we reasoned that given the results from siRNA knockdown and *in vitro* digestion experiments, it was not necessary to test further inhibitors. Taken together, the results of small molecule inhibitors and siRNA knockdown indicate that both cathepsin L and cathepsin B are possibly involved in ABCA3 processing. However, the moderate effect of CA-074Me and the *in vitro* digestion of ABCA3 peptide indicate that the activity of cathepsin B towards ABCA3 is much lower than that of cathepsin L. It is thus questionable whether cleavage of ABCA3 by cathepsin B is physiologically relevant.

Cleavage of ABCA3 is a further example for a highly specific function of cathepsins. While they were long believed to perform unspecific bulk proteolysis, it has now become evident that cathepsins also carry out specific non-redundant *in vivo* functions [24]. It has also been discussed that expression of ubiquitous cathepsins in specialized cells—which certainly applies to alveolar epithelial type II cells—may indicate a highly specific function for a rather nonspecific protease [25]. Indeed, cathepsin L was shown to be involved in the processing of cathepsin D in A549 cells [26]. There are a few examples of lysosomal membrane proteins as cathepsin L substrates [27]. In the case of cathepsin L, its involvement in the processing of DIRC2 and CLN7 has been demonstrated [18,28]. CLN7 was the first example of a disease-associated lysosomal membrane protein that is processed by cathepsin L [18].

Analysis of peptide fragments using mass spectrometry showed that the peptide was cleaved by cathepsin L after Lys^174^ in concordance with the cleavage site we had found for full-length ABCA3 in living cells. For cathepsins, it was found that S2 is the primary specificity-defining site [29]. The respective residue in the case of ABCA3 would be Leucine which is accepted at this position by most lysosomal cysteine proteases. Indeed, Leu^173^ and Lys^174^ seem to be important for the recognition by cathepsin L as exchanging both residues for alanine resulted in a marked inhibition of cleavage (Fig 7). The observation of *in vitro* cleavage by cathepsin L also implies that ABCA3 is a direct substrate of cathepsin L rather than of a downstream protease activated by cathepsin L.

Cathepsin L deficiency in mice is not associated with lung disease [24]. Although it cannot be concluded that the same is necessarily true in humans, no lung disease associated with mutations in the cathepsin L gene has been observed so far. Thus, it seems that cathepsin L-mediated cleavage is not critically required for functional activation of ABCA3. This would support the hypothesis that full-length ABCA3 is functionally active and that proteolytic cleavage serves to regulate ABCA3 levels rather than activating the transporter. However, it can be expected that a certain level of ABCA3 is needed for sufficient biosynthesis of lamellar bodies and secretion of surfactant into the alveolar space. Since the amount of ABCA3 depends on the rates of synthesis and degradation, it is reasonable to speculate that disturbances of the steady state may negatively affect surfactant homeostasis and ultimately lead to disease. On the other hand, when ABCA3 degradation was inhibited by targeting cathepsin L activity, a positive effect on ABCA3 activity could be expected. Interfering with the activity of cathepsin L in alveolar epithelial type II cells may thus represent an approach to ameliorate ABCA3 function in patients suffering from lung disease due to ABCA3 haploinsufficiency [30].
